# The tobacco chloroplast *YCF4* gene is essential for transcriptional gene regulation and plants photoautotrophic growth

**DOI:** 10.3389/fpls.2022.1014236

**Published:** 2022-10-24

**Authors:** Muhammad Sarwar Khan, Rimsha Riaz, Muhammad Majid, Kashif Mehmood, Ghulam Mustafa, Faiz Ahmad Joyia

**Affiliations:** ^1^ Center of Agricultural Biochemistry and Biotechnology (CABB), University of Agriculture, Faisalabad, Pakistan; ^2^ Department of Molecular and Cellular Biology, Summerlee Science Complex, University of Guelph, Guelph, ON, Canada

**Keywords:** *YCF4*, heterotrophic, knockout, transcript analysis, null alleles, homoplasmic

## Abstract

A tobacco chloroplast hypothetical open reading frame 4 (YCF4) has been reported as a non-essential assembly factor for photosynthesis based on an incomplete knockout of YCF4, just 93 of 184 amino acids from the N-terminus were knocked out. On the other hand, we removed the complete sequence of *YCF4* from tobacco chloroplasts and observed that Δ*YCF4* plants were unable to survive photoautotrophically as their growth was hampered in the absence of an external carbon supply, clearly showing that the YCF4 is essential for photosynthesis. Initially, the *aadA* gene was introduced into the tobacco plastome replacing the complete *YCF4* gene through homologous recombination events. The replacement of *YCF4* with *aadA* was confirmed by PCR and Southern blot analysis in Δ*YCF4* plants. Homoplasmic Δ*YCF4* plants had a light green phenotype, and the leaves became pale yellow as the plants grew older. The structure of chloroplasts of Δ*YCF4* mutants of light green phenotype was studied using a transmission electron microscope (TEM), and the micrographs demonstrated structural anomalies in the chloroplasts; including shape, size, and grana stacking compared to the wild-type plants. Further, transcriptome analysis revealed that the expression of PSI, PSII, and ribosomal genes remained unchanged in ∆*YCF4* plants. On the other hand, transcriptome levels of *rbcL* (Ribulose 1,5-bisphosphate carboxylase/oxygenase large subunit), *LHC* (Light-Harvesting Complex), and *ATP Synthase* (*atpB* and *atpL*) decreased, indicating that the YCF4 has the function(s) in addition to assembling the photosynthetic complex. This was confirmed by *in-silico* protein-protein interactions of full-length YCF4 as well as 93 and 91 of 184 amino acids from N- and C-termini of the full-length protein, which revealed that the C-terminus (91 aa) of YCF4 is important in interacting with other chloroplast proteins. These findings provide genetic support for the plastid *YCF4* gene’s critical role in regulating the plastid gene expression and assembling the photosynthetic complex.

## Introduction

Plastid origin can be traced back over a billion years when a fossil red alga was discovered, providing substantial proof for the theory (Embley and Martin, 2006; Song et al., 2018) that plastids arose from endosymbiotic photosynthetic bacteria (Schimper, 1883). Plastids are the defining traits of plants and green algae, yet they maintain many prokaryotic properties. The bacterial genome has reduced dramatically over time, owing to gene loss and large-scale gene transfer to the nuclear genome. Thus, plastid genomes referred to as ‘plastomes’ contain around 120-130 genes, the majority of which encode components of the organelle’s gene expression mechanism and photosynthetic apparatus and are structured in nucleoids. Plastids, on the other hand, have far more proteins than their plastomes can code for. As a result, most plastid proteins are now encoded by the nuclear genome and must be transported into the organelle after translation. Plastids have an extensive membrane system, thylakoids, which are condensed to form, grana, in addition to two envelope membranes. In thylakoid membranes of plants including cyanobacteria and algae two enormous multimeric chlorophyll-binding protein complexes, photosystems I and II are embedded that performs the first step in the oxygenic photosynthesis this process, responsible for converting sunlight into chemical energy (Blankenship, 2010; Kargul and Barber, 2011).

The plastid genome was first sequenced in tobacco followed by hundreds of higher plants that have been sequenced and characterized. This plethora of plastid genome data insights researchers for the functional characterization of plastid-encoded genes. Most of the chloroplast genes have been characterized for their role in the organellar stability or metabolic activities within the chloroplasts, yet few of these are still to be worked out for their function. They are labeled as ycfs (hypothetical chloroplast open reading frames). Few of these have been characterized as non-essential genes whereas others as essentials. The YCF4 gene, one of these ycfs, is a highly conserved protein in cyanobacteria, green algae, and land plants. Previously, it was discovered that the *YCF4* gene product is involved in the formation of the PSI complex in *Chlamydomonas reinhardtii* (Boudreau et al., 1997). In *YCF4* mutants, PSI activity was completely lost, resulting in autotrophic growth failure. Inactivation of the *YCF4* homolog causes an increase in the PSII-to-PSI ratio in the cyanobacterium *Synechocystis* sp. PCC 6803. (Wilde et al., 1995). The thylakoid membrane-intrinsic YCF4 was found in complexes with the PSI subunits PsaA through PsaF and the opsin-related eyespot protein COP2 in Chlamydomonas (Ozawa et al., 2009). Because RNA interference did not influence PSI accumulation in the alga, the COP2 protein is unlikely to play a role in PSI biogenesis (Ozawa et al., 2009). YCF4 has also been found as a protein component of the eyespot in Chlamydomonas chloroplasts (Schmidt et al., 2006), suggesting that this has a second function in the eyespot (in conjunction with COP2). Furthermore, utilizing reverse genetics, *YCF4* has been knocked out in tobacco, and the mutants have shown autotrophic growth (Krech et al., 2012). Tobacco *YCF4* knockout mutants were able to assemble enough PSI to enable modest autotrophic growth. However, the *YCF4* mutants did not grow autotrophically in our investigations, and extremely slow growth was detected under controlled settings using sucrose as a carbon source, but not in wild conditions.

In the present studies, we have removed the complete *YCF4* gene sequence encoding 184 amino acids and developed homoplasmic Δ*YCF4* plants, exhibiting very slow growth on an artificial medium supplemented with varied sucrose levels. These mutants unlike previously reported *YCF4* mutants (Krech et al., 2012) where the partial sequence of *YCF4* encoding 93 amino acids from the N-terminal region of YCF4 has been removed leaving 91 amino acids of the C-terminal intact, failed to grow autotrophically under normal conditions in peat moss-containing pots. It was observed by *in-silico* protein-protein interactions of full-length YCF4 as well as 93 and 91 of 184 amino acids from N- and C-termini, respectively of the full-length protein that the C-terminus (91 aa) of YCF4 is interacting with other chloroplast proteins.

## Materials and methods

### Plant material for targeted knockout of *YCF4*

N*icotiana tabacum* L. var. Petit Havana was grown at 25 ± 1˚C under 16 hrs light (white light: 100 µmol·m^-2^·s^-1^) and 8 hrs dark regime in a growth room. The sterilized seeds were cultured on RMOP medium containing MS salts (4.33 g/L), Myoinositol (100 mg/L), BAP (1.0 mg/L), NAA (0.1mg/L) sucrose (3%) solidified with 0.026% Gelrite. Fully mature dark green leaves of 4-6 weeks old plants were used for targeted knockout of the *YCF4* gene (Nazir and Khan, 2013).

### Development of chloroplast transformation vector and selection of putative knocked out plants

Considering the location of the *YCF4* gene where *rbcL, accD*, and *psaI* are in the upstream region and *ycf10*, *petA*, and *psbJ* are in the downstream region of the *YCF4*. To develop chloroplast transformation vector for the targeted inactivation of *YCF4*, *ycf10* sequence was cloned as right border flanking sequence whereas *PsaI* along with few nucleotides of *accD* were cloned as left border flanking sequences. FLARE-S cassette having *aadA* (aminoglycoside 3́-adenyltransferase) and *gfp* (green fluorescent protein) was cloned in between the plastid flanking sequences (Khan and Maliga, 1999). The resultant plastid transformation vector was coated on 0.6 μm gold particles and were bombarded on tobacco leaves using particle gun (Bio-Rad, USA). The bombarded leaves were chopped into tiny slices and cultured on RMOP medium containing 500 mg/L spectinomycin. Antibiotic resistant shoots were rooted on MS medium containing 30 g/L sucrose.

### Genomic analysis of putative transplastomic clones for targeted inactivation of *YCF4*

Total cellular DNA was isolated from the transformed and untransformed tobacco plants using CTAB (hexadecyltrimethyl ammonium bromide) method with certain modifications. The isolated DNA was subjected to PCR (Polymerase Chain Reaction) to confirm the transgene integration using primers flanking *aadA* selectable marker gene (A19: 5’-GGC TCC GCA GTG GAT GGC GGC CTG-3’; A20: 5’-GGG CTG ATA CTG GGC CGG CAG G-3’) Further, the homoplasmic status of transplastomics were accessed using S19 and S20 primers flanking *psaI* and *ycf10* genes. Following PCR profile was used for the amplification of the above-mentioned gene(s): 94°C for 1.5 minutes 56°C for 1.0 minutes and 72°C for 3 minutes with a final extension at 72°C for 10 minutes and a total of 35 cycles.

Homoplasmy of the *YCF4* knock-out plants was confirmed by Southern blot analysis. A total of 13 μg genomic DNA was digested with the restriction enzyme *Bam*HI restriction endonuclease and was resolved on 1% agarose gel. After washing with depurination, denaturation, and neutralization solutions, the restricted DNA was blotted onto a nitrocellulose membrane. Thereafter, it was hybridized with a biotin-labeled probe followed by detection with a chromogenic detection kit (Thermo scientific, USA).

### Transcript analysis of *∆YCF4* plants

Total cellular RNA was isolated from the confirmed purified ∆YCF4 and wild type *in vitro* growing tobacco plants by RNeasy Plant Mini Kit (Qiagen, USA). The isolated RNA was treated with DNase I (Thermo Fisher Scientifc, USA) to remove genomic DNA following the manufacturer’s instructions. RevertAid First Strand cDNA synthesis kit (Thermo Fisher Scientific, Cat. # K1621) was used for the synthesis of cDNA using 1.0 µg RNA following the manufacturer’s instructions. The resultant cDNA was used for semi-quantitative expression analyses of *psaA*, *psaB*, *psaC*, *psaH* (PhotosystemI), *psbA, psbB, psbC, psbD and psbE* (Photosystem II), ATP synthase, *rps16*, *rps2*, *rrn16*, *ycf10*, *rbcL clpP*, *rpoA*, and *rpoB*.

### Electron microscopy to study chloroplast ultrastructural variation in *∆YCF4* plants

Transmission Electron Microscopy (TEM) was carried out following the protocol given by Islam et al., 2008. Leaves from ∆YCF4 and wild-type tobacco plants were cut into 1 to 2 mm^2^ pieces and fixation was carried out using 0.2 M phosphate buffer (PBS, pH 7.2) containing 4.0% glutaraldehyde (v/v) at 4°C for 6-8 hours. Postfixation of samples was carried out in 1% osmium tetroxide (OsO_4_) for 1 h at 4°C and then incubated in 0.2M PBS (pH 7.2) for 1-2 hours at room temperature. The dehydration was carried out using a graded series of ethanol and acetone and finally, the leaf pieces were embedded in Spurr’s resin. Ultrathin sections (~70 nm) of embedded leaf samples were prepared on an ultramicrotome (RMC Mt 7000) and mounted on copper grids to be viewed in the TEM (JEOL, Model JEM-1010) at an accelerating voltage of 90.0 kV. Multiple images of each section were recorded by exposing a photographic film and later developed in the darkroom.

### Determination of physiological parameters

Various photosynthesis-related physiological parameters were determined using IRGA (Infrared gas analyzer). The physiological parameters included photosynthetic rate, transpiration rate, substomatal conductance, substomatal CO_2,_ and light intensity.

### Heterotrophic/autotrophic nature of growth of *YCF4* mutants

To assess the impact of carbon starvation on the growth, mutant as well as wild-type tobacco plants were cultured on MS medium augmented with different levels of carbon (0, 0.5, 1.0, 1.5, 2.0, 2.5, and 3.0% sucrose). The plants were grown in normal light. Data were recorded to see the impact of carbon starvation on mutant tobacco plants.

### Molecular docking of YCF4 protein with other photosynthesis-related proteins

The molecular docking of full-length and truncated versions of YCF4 with other photosynthesis-responsive proteins was performed using an online web server ClusPro 2.0. It predicts the interaction between the candidate proteins through rigid-body docking based on Fast Fourier Transform, followed by clustering, and minimizing the docked complex to get the highly populated cluster having the least-energy conformation (Kozakov et al., 2017). The first 93 amino acids of the YCF4 were knocked out by Krech et al., 2012 in their study leaving 91 amino acids intact. Whereas, we have used 93 amino acids from the N-terminal and 91 amino acids from the C-terminal side of the YCF4 as well as full-length YCF4 for studying their interaction with YCF10, ribosomal proteins or RNA (rps16, rps2, rrn16), subunits of PS-I (psaA, psaB, psaC, psaH), subunits of PS-II (psbA, psbB, psbC, psbD, psbE), alpha and beta chains of ATP synthase (atpB, atpI) and other photosynthetic proteins (rbcL, clpP, rpoA, rpoB, accD, petA, Light-harvesting complex (LHC) using ClusPro. The docked complexes with the maximum clustering members and minimum energy scores were selected. DIMPLOT program of Ligplot+ v.4.5.3 was used to find the number of hydrogen bonds and the bond lengths between the interacting residues (Wallace et al., 1996).

## Results

### Development of *YCF4* mutants and their purification to homoplasmic level

**Figure 1:**
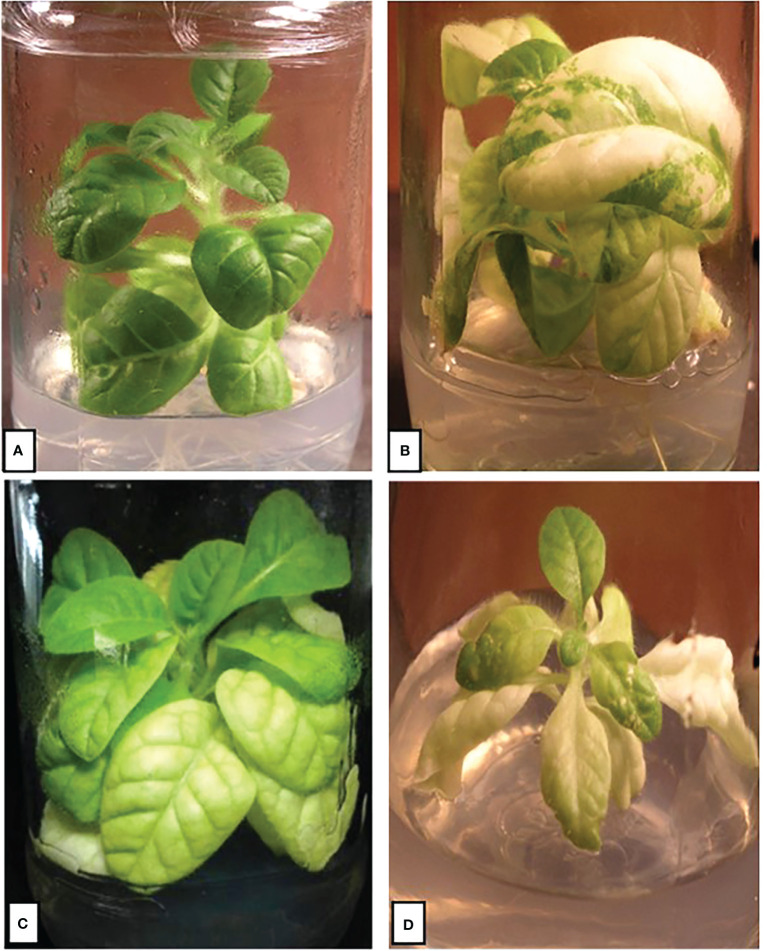
Purification of *Δycf4* plants on spectinomycin-containing selection and regeneration medium. **(A)** Wild-type tobacco plants with intact *ycf4* showing normal growth with lush green leaves. **(B)** Heteroplasmic plant showing leaves carrying chimeric tissues with both knocked-out and wild-type cells. **(C)** Homoplasmic *Δycf4* tobacco plant growing under low light. **(D)** Homoplasmic *Δycf4* tobacco plant growing under normal light.

**Figure 2:**
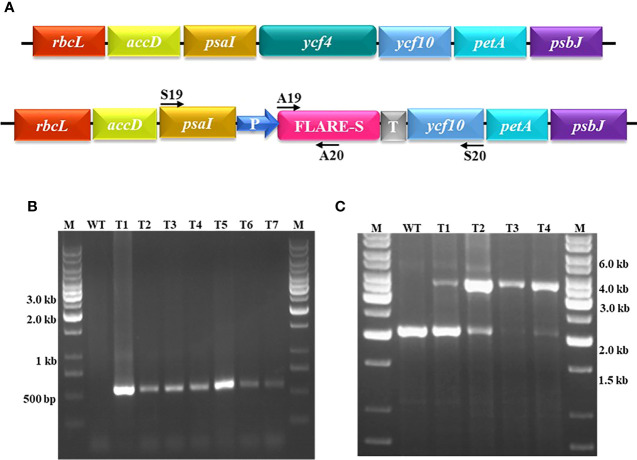
Confirmation of transgene integration into plastid genome and determining the plasmy level by Polymerase Chain Reaction. **(A)** Physical map showing the positions of the primers used. **(B)** Entire pool of spectinomycin-resistant plants regenerated on regeneration medium were screened for the presence of marker gene aadA-specific primer set (A19/A20): M is 1 kb marker DNA, WT is the untransformed tobacco, and T1-T7 are putative transplastomic plants. **(C)** Out of screened plants four as T1-T4 were randomly selected for the determination of plasmy level using S19/S20 primer set that lands on flanking sequences: M represents 1.0 kb DNA ladder, WT represents wild-type tobacco plant whereas T1-T4 represents deletion level of *ycf4* from the chloroplast transgenic plants regenerated on spectinomycin-containing regeneration medium. Amplification of a fragment of 4.0 kb indicates transgene integration whereas amplification of a fragment of 2.0 kb represents wild-type plastid DNA.

**Figure 3:**
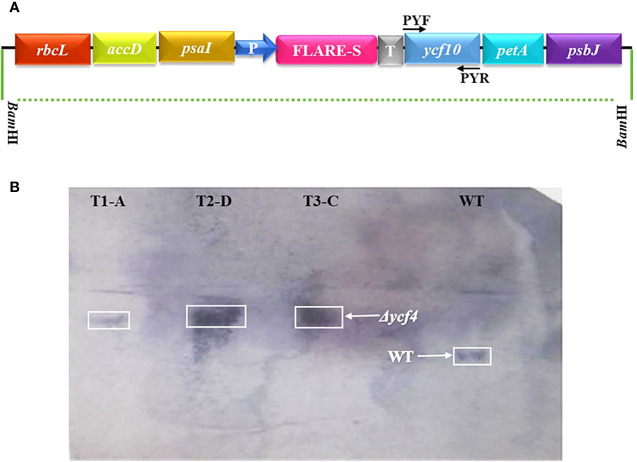
Confirmation of plasmy level of *Δycf4* plants through Southern Blot Analysis. **(A)** Physical map showing location of primers used to amplify the probe. **(B)** Total cellular DNA of *Δycf4* and wild-type tobacco plants was digested with *Bam*H1, transferred onto the nitrocellulose membrane, and hybridized against a biotin-labeled probe prepared from ycf10 that amplifies both transformed and wild-type chloroplast DNA since ycf10 gene-specific probe detects all plastome copies irrespective to their genotype whether transformed or wild-type (untransformed). However, these plants were selected from an already screened pool (T1-T4 as shown in Figure 2C) of transgenic plants.

The *YCF4* mutants were developed by targeting the FLARE-S (*aadA* and *gfp*) cassette with flanking sequences of *ycf10* and *psaI*. Sequential amplification and cloning of *ycf10* as right border flanking sequence and *PsaI* together with a few nucleotides of *accD* as left border flanking sequence resulted in the development of a plasmid transformation vector. The FLARE-S and its regulatory sequences were cloned in between the plastid flanking sequences. The resulting cassette was inserted into the plastid genome to inactivate *YCF4* specifically. Leaf sections were cultivated in an RMOP medium containing a selective agent, spectinomycin (500 mg/L), after particle bombardment. The antibiotic-resistant shoots were putative transformants, but they were indistinguishable from untransformed tobacco plants growing in comparable conditions. For the purification of transplastomic cells, they were exposed to additional rounds of selection and screening. Leaves were chopped into small pieces and cultured on the selective agent for this purpose. All of the plants had a light green to yellow phenotype after several rounds of continuous selection and screening, showing homotransplasmicity (**Figure 1**). Antibiotic-resistant shoots were validated using various primer sets. To validate the integration of *transgene* and deletion of *YCF4*, putative transgenic plants were tested using primers flanking the *aadA* gene and the deletion cassette (**Figure 2A**). The incorporation of the *aadA* into the targeted genome was confirmed by amplification of a 552 kb fragment (**Figure 2B**). Furthermore, *YCF4* knockout was validated using primers flanking the gene with no amplification like wild-type plant samples. Between *psaI* and *ycf10*, amplification of a 2.0 kb fragment from wild-type tobacco plants and a 4.0 kb fragment from *ΔYCF4* plants demonstrated transgene incorporation into the plastid genome (**Figure 2C**). The amplification of 2kb fragment in transformants indicates that the transgenic plants are heteroplasmic, and the *YCF4* has not been deleted completely from the plastome. The possibility of selecting nuclear transformants, spontaneous mutants (owing to mutations in the 16SrRNA gene), or escapes was eliminated. The plasmy level of the selected transformed shoots was also confirmed by Southern Blot Analysis (**Figure 3A**). The purified cellular DNA was restricted with *Bam*HI and transferred onto blotting membrane. The presence of a single large-size hybridizing fragment of ~4.0 kb confirmed the homoplasmy of the FLARE-S integration and *YCF4* gene deletion (**Figure 3B**) from the tobacco plants.

### Impact of *YCF4* deletion on phenotype and mode of nutrition

**Figure 4:**
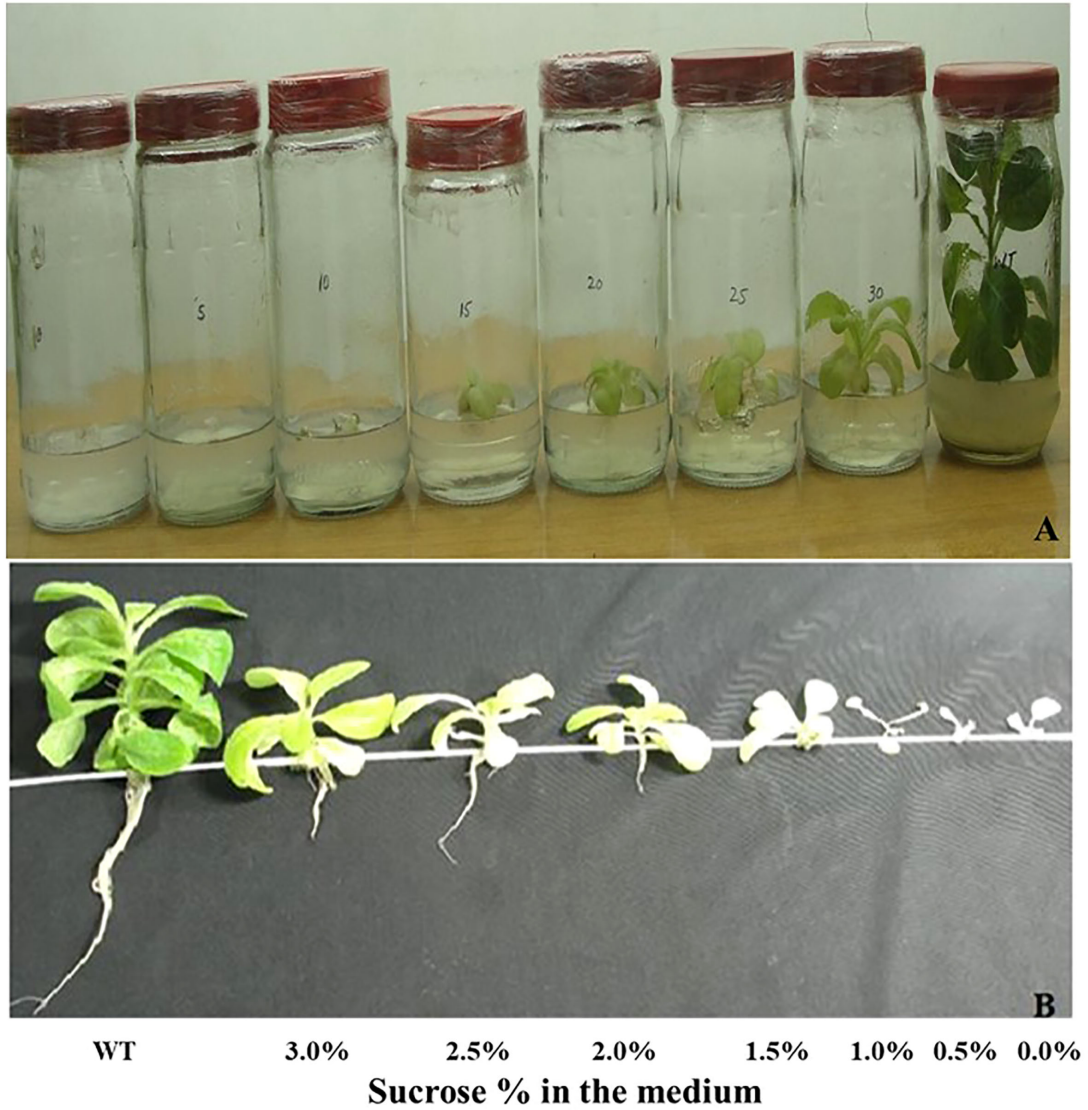
** (A)** Homotransplastomic *Δycf4* tobacco plants were cultured on different levels of sucrose (0.0%, 0.5%, 1.0%, 1.5%, 2.0%, 2.5% and 3%) to assess autotrophic/heterotrophic nature of *Δycf4* plants in comparison with wild-type *in vitro* growing tobacco plants. **(B)** Plants at A were taken out of the jars.

Homoplastic ∆*YCF4* plants appeared to have a distinct phenotype. The newly emerged younger leaves were green which gradually bleached out with the attainment of maturity. Lowermost leaves were almost white with minimum chlorophyll whereas top-most leaves were green with comparatively higher content of chlorophyll when plants were grown under normal light. The leaves continued to bleach until the whole plant was discolored and showed stunted growth with an inability to grow autotrophically. To eliminate the possibility that standard light (60 µmol m-2 s-1) may cause photobleaching, the plants were grown under low light (30 µmol m-2 s-1), and a light green phenotype was maintained in the ∆*YCF4* plants. Further, to assess the impact of the autotrophic mode of nutrition, plants were grown at different levels of sucrose (0, 0.5, 1.0, 1.5, 2.0, 2.5, and 3.0%). Mutant plants cultured on 0, 0.5, and 1% sucrose were unable to survive and no leaf development was observed. However, plants cultured on 1.5, 2, 2.5, and 3.0% sucrose showed growth, and leaf development was observed with increased sucrose concentration. The ∆*YCF4* plants cultured on 3% sucrose were light leaves green in color compared with the wild-type plants which were green in color with normal growth (**Figure 4**). Further, several mutant plants growing on 3% sucrose were shifted to compost-containing pots for acclimatization, but they were unable to survive. This confirmed that *YCF4* mutants are unable to survive autotrophically rather they require an additive carbon source for sustainability and growth.

### Electron microscopy revealed ultrastructural variations between normal and *∆YCF4* plants

**Figure 5:**
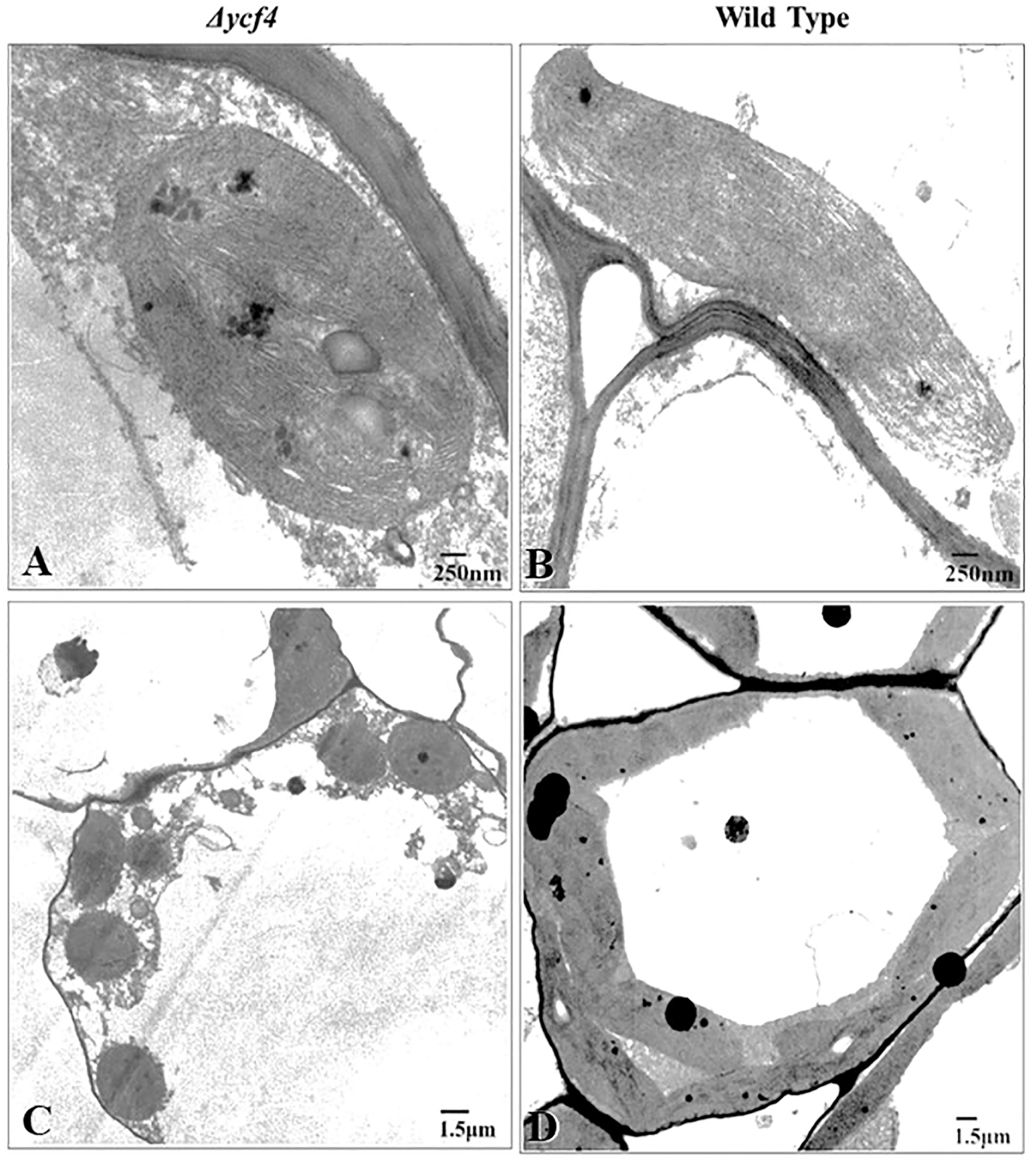
Transmission electron microscopy of *Δycf4* and untransformed tobacco leaves shows the presence of defective plastids in the leaves of bleached plants. **(A, B)** Chloroplasts in light green leaves of *Δycf4* plants **(C, D)** Chloroplasts in lush green leaves of wild-type plants. The micrographs showed that *ycf4* knockout caused structural anomalies including smaller size and rounded shape of chloroplasts in leaves.

To investigate the ultrastructural variations in chloroplasts due to the absence of YCF4, leaf tissues from *∆YCF4* mutant and wild-type plants were analyzed using a transmission electron microscope (TEM). Ultrastructural studies revealed that chloroplasts in knockout plants underwent substantial structural changes that may be correlated with the absence of YCF4 protein. **Figure 5** revealed distinct variations in chloroplast size and shape between knockout plants and wild-type (normal) plants. The TEM results showed that chloroplasts in wild-type plants were oblong in shape and larger in size than those of mutant knockout plants which were almost rounded. Further, the thylakoid membranes were densely packed in chloroplasts of wild-type plants as compared to those of knockout plants. In knockout plants, the grana thylakoids were less discrete, and their stacks exhibited a loss of their orderly structure. As the thylakoid membranes became less organized some vesicular structures appeared in mutant chloroplasts (**Figure 5A**).

### Physiological and photosynthetic performance of *YCF4* mutants

**Figure 6:**
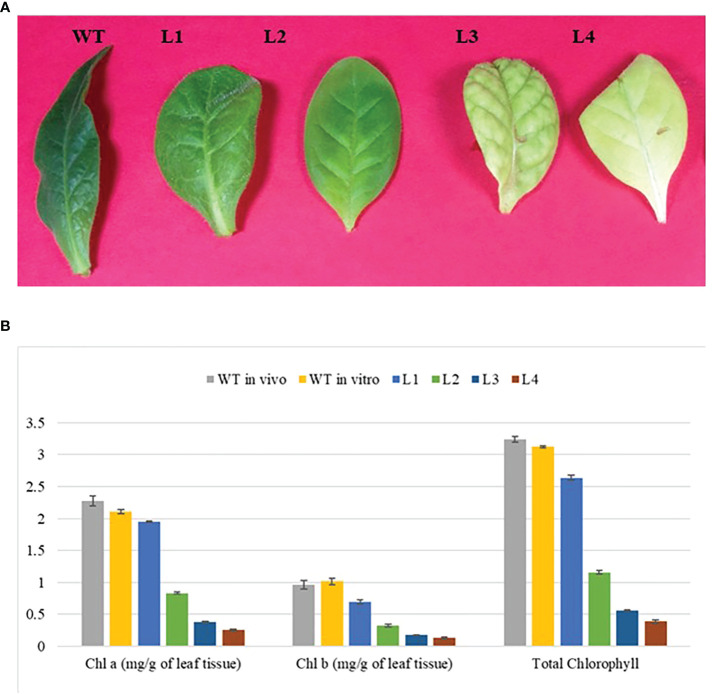
Photosynthetic performance of *∆ycf4* plants. **(A)** Leaves samples used for chlorophyll estimation **(B)** Graphical representation showing chlorophyll content in wild-type and transgenic plants. The lower contents of photosynthetic pigments in the *∆ycf4* plants indicate that they were physiologically incompetent as compared with wild-type tobacco plants. WT represents wild-type whereas L1, L2, L3, and L4 represent 1st, 2^nd^, 3rd and 4th leaf of ***∆*** *ycf4* plants from top to bottom, respectively.

**Figure 7:**
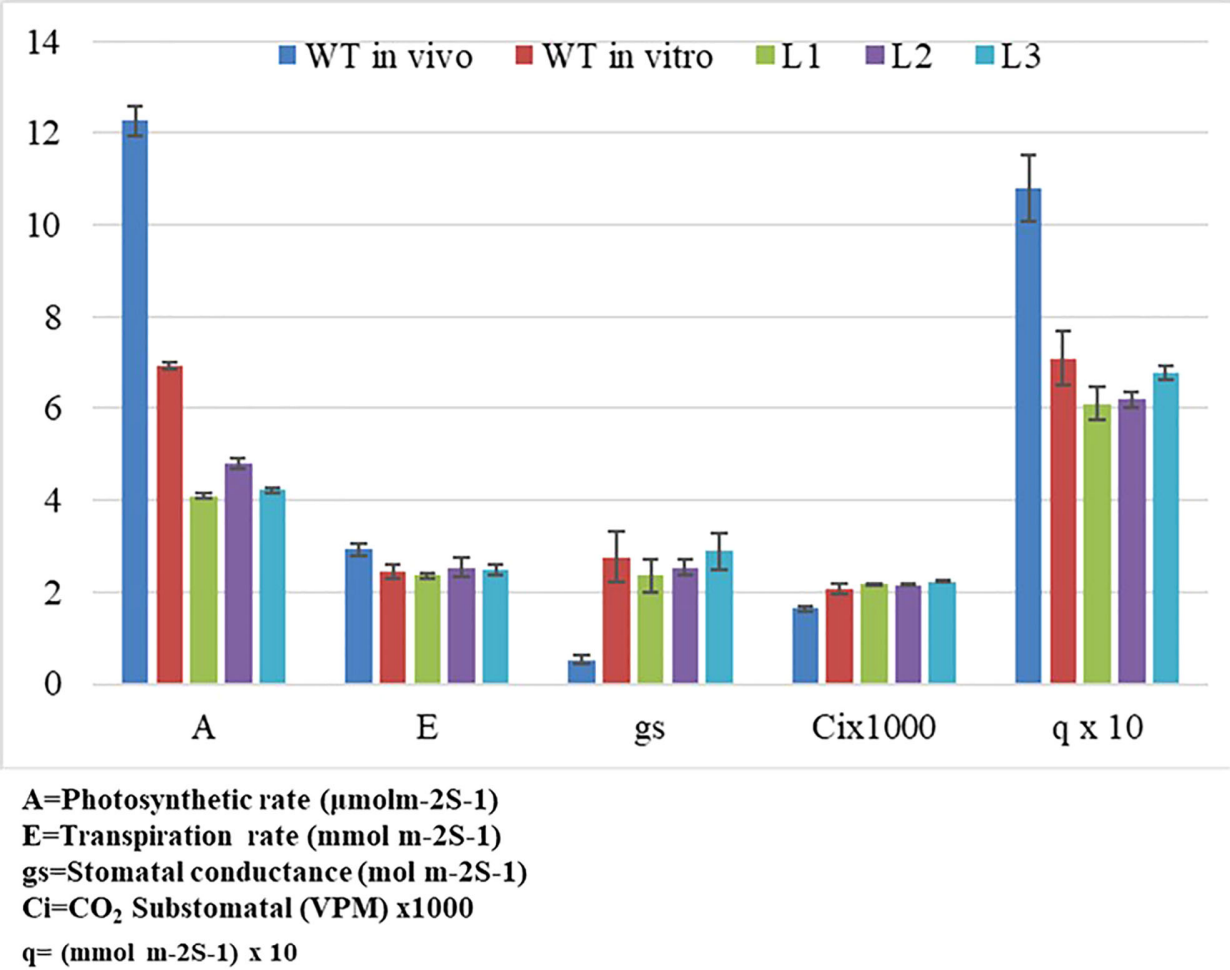
Impact assessment of *ycf4* deletion on physiological performance of transplastomic *∆ycf4* and wild-type (WT) tobacco plants. WT represents Wild-type whereas L1, L2, and L3 represent 1st, 2^nd^, and 3rd leaf of *∆ycf4* plants from top to bottom, respectively.

The *YCF4* gene deletion appeared to have profound effects on the photosynthetic and physiological performance of mutant plants (**Figure 6A**). The mutants were unable to attain normal contents of total chlorophyll as the topmost young leaves of mutant plants accumulated 2.6 mg/g of chlorophyll compared to the wild-type plant leaves (3.1 mg/g). The levels were decreased up to 99.98% in non-photosynthetic cells of mutants as the plant matures, from top to bottom (**Figure 6B**). Likewise, physiological parameters including photosynthetic rate (A), transpiration rate (E), stomatal conductance (gs), sub-stomatal CO_2_ (Ci), and photosynthetic photon flux density (q also lux) also revealed that *YCF4* plants were physiologically incompetent as compared with normal tobacco plants (**Figure 7**).

### Transcript analysis of plastid-encoded genes in *∆YCF4* plants

**Figure 8:**
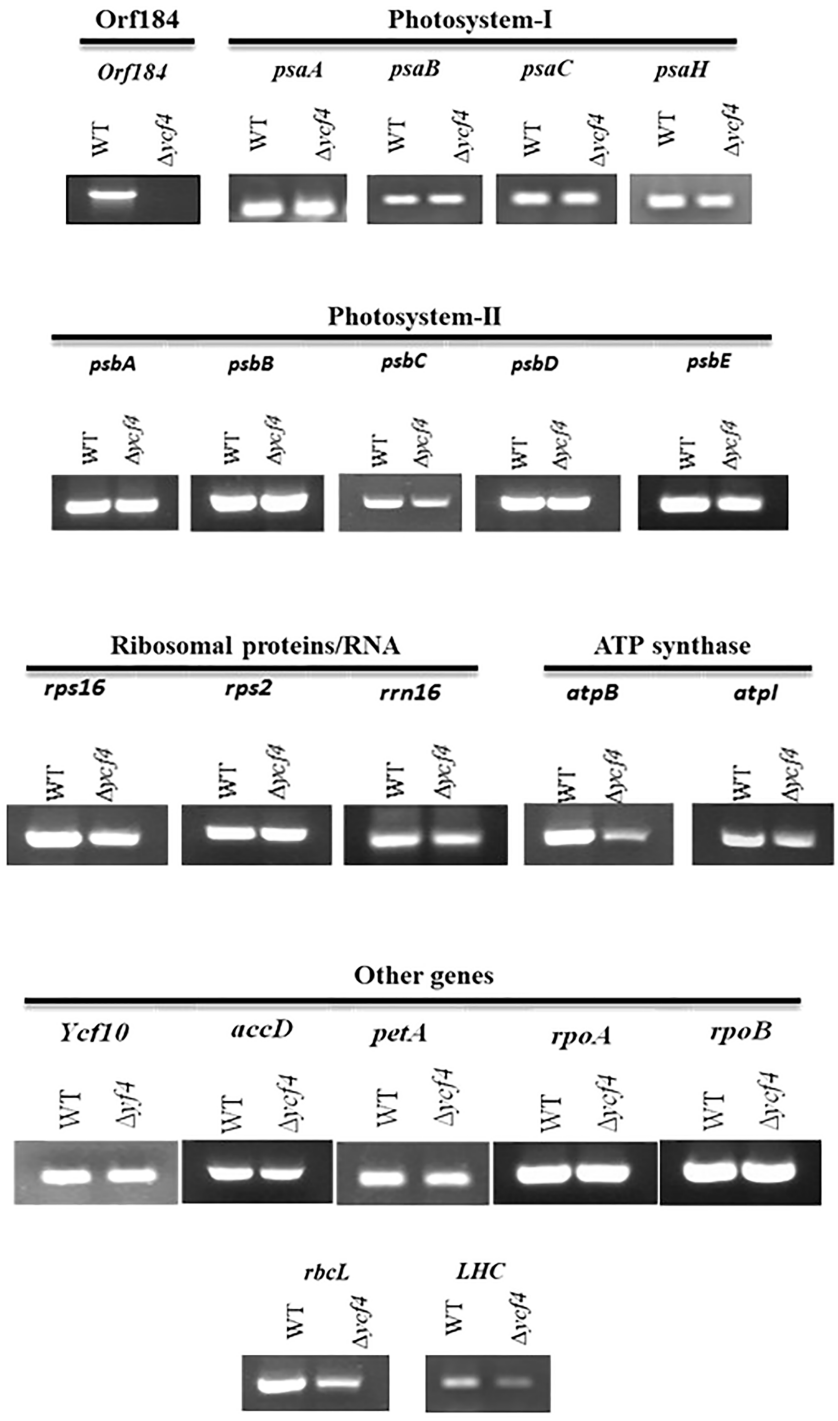
Transcript analysis of *∆ycf4* and wild-type tobacco plants. Transcripts show the expression level of genes in both wild-type and *∆ycf4* plants. The expression of various subunits of photosystem-I (psaA, psaB, psaC, psaH) and photosystem-II (psbA, psbB, psbC, psbD, psbE) remained unaltered. The absence of a transcript in *∆ycf4* indicates the purification of the *∆ycf4* plants to homoplasmy.

The effect of *YCF4* deletion on the expression of plastid-encoded genes was determined by transcript analysis of mutant plants. No transcripts were detected in purified *∆YCF4* plants since the gene has been completely replaced with a marker gene. The transcripts levels of *psaA, psaB, psaC* and *psaH* encoding photosystem-I proteins appeared the same in the ∆*YCF4* plants and in normal untransformed tobacco plants. Likewise, the accumulation of *psbA, psbB, psbC, psbD, psbE* transcripts encoding Photosystem-II proteins were not decreased significantly in these mutants suggesting that *YCF4* deletion did not have a direct role in the transcriptional regulation of PS-I and PS-II genes. Further, the transcript levels of ribosomal protein-encoding genes *rps16*, *rps2*, and *rrn16*, and of *ycf10, rpoA, rpoB, aacD* and *petA* remained unchanged. Interestingly, the transcript levels of *rbcL* and *lhc* genes were significantly reduced, suggesting that *YCF4* deletion affects the accumulation of RUBISCO and LHC1, eventually the photosynthesis (**Figure 8**).

### YCF4 interaction with other photosynthesis proteins

The molecular interaction of full-length YCF4 with PS-I subunits indicated strong interaction with psaB, psaC, and psaH, each having seven hydrogen bonds with YCF4. But YCF4+psaC complex showed the most stable interaction among them with bond lengths of 2.62-2.93Å (**Supplementary Figure S1**). The bond length corresponds to the strength of interaction between interacting molecules and should be <4Å as the greater the bond length, the weaker will be the interaction. Likewise, among the PS-II subunits psaE showed a peculiar binding pattern and formed five hydrogen bonds with YCF4 (**Supplementary Figure S2**). The ATP synthase consists of two chains namely the alpha chain and beta chain. Both chains are known to interact with YCF4 to carry out photosynthesis (Yamori et al., 2011). Only the beta chain (atpB) revealed an effective docking pattern with YCF4 forming twelve hydrogen bonds with a bond length of 2.56-3.15Å (**Supplementary Figure S3**). Ribosomal proteins were also investigated as they are reported to play an essential role in the development of plant phenotypes (Horiguchi et al., 2012). rrn16 showed a strong affinity with YCF4 protein forming ten hydrogen bonds within the bond length range of 2.63-3.19Å. Likewise, the interaction of YCF4 with other plastidic proteins was also determined that showed the maximum binding affinity with rbcL among all the candidate proteins.

These photosynthetic proteins were further docked with carboxylic and amino terminus of YCF4 protein to elucidate which portion of the protein is more essential for photosynthesis. Among PS-I proteins, the amino terminus of YCF4 revealed maximum interaction with psaB forming five hydrogen bonds while twelve hydrogen bonds were observed in the carboxyl terminus of the YCF4+psaH complex. The psbE of PS-II bonded with the amino terminus of YCF4 effectively as compared to other proteins but formed only six hydrogen bonds while psbC strongly docked making thirteen hydrogen bonds with the terminus of YCF4. The carboxyl terminus of YCF4 also demonstrated durable interaction with other PS-II proteins but weaker than psbC. The docked complexes of the carboxyl terminus of YCF4 with rps2, rps16 and rrn16 showed 7, 11, and 6 hydrogen bonds respectively, while the amino terminus of YCF4 bonded with these proteins comparatively stronger showing 14, 18, 18 hydrogen bonds with rps2, rps16 and rrn16 (**Supplementary Figure S4**). Furthermore, the beta chain of ATP synthase is supposed to interact better compared to the alpha chain. The beta chain formed only eight hydrogen bonds with the amino terminus of YCF4 however twenty-eight hydrogen bonds were found in the carboxyl terminus of YCF4+atpB (**Supplementary Figure S3**). Likewise, assessing other proteins, rpoB found to be well-interacted with the truncated versions of YCF4 than other proteins revealing nine bonds with amino terminus and twenty-five bonds with carboxyl terminus of YCF4 (**Supplementary Figure S5**).

The YCF4 was also docked with the four core subunits of LHC of PS-I and a nuclear-encoded small subunit (RBCS) of RUBISCO to further validate the findings. Their carboxyl terminus showed stronger interaction with a high number of hydrogen bonds forming between them. The relevance of the carboxyl terminus of YCF4 is once again demonstrating its strong connection with LHCA1, LHCA2, LHCA3, LHCA4, and RRBC (**Table 1**). The number and length of hydrogen bonds in the docked complexes of full-length and truncated YCF4 variants with many other proteins are shown in **Table 1**. Thus, these *in-silico* investigations support the current study’s notion that the carboxylic terminus of YCF4 is more crucial for photosynthesis than the amino terminus.

**Table 1:** Number of hydrogen bonds and the range of bonds length in docked complexes.

		Hydrogen	Bond length	Hydrogen	Bond length	Hydrogen	Bond length
	Docked complex	bonds (full- length	range	bonds (amino terminus of	range	bonds (carboxyl terminus of	range
		**YCF4**		**YCF4**		**YCF4**	
	ycf4+psaA	3	2.85 - 3.07	3	2.84-2.93	5	2.74-3.02
	ycf4+psaB	7	2.58 - 3.22	5	2.75-2.89	12	2.61-3.20
	ycf4+psaC	7	2.62 - 2.93	1	2.50	8	2.62-3.19
**PS-I**	ycf4+psaH	7	2.57 - 3.01	4	2.73-3.05	17	2.57-3.26
	ycf4+LHC	3	2.62 - 2.91	4	2.72-2.85	9	2.83-3.20
	ycf4+LHCA1	5	2.03 - 2.76	4	2.35-3.76	9	2.45-3.76
	ycf4+LHCA2	1	2.87 - 3.22	3	2.78-3.43	12	2.01-2.92
	ycf4+LHCA3	4	2.56 - 3.48	6	2.46-3.88	10	2.64-3.67
	ycf4+LHCA4	4	2.09-3.22	5	2.55-2.76	9	2.67-3.66
**PS-II**	ycf4+psbA	4	2.73 - 3.21	3	2.67-3.15	8	2.73-3.28
	ycf4+psbB	4	2.73 - 3.05	2	3.04-3.08	6	2.47-3.07
	ycf4+psbC	2	2.77 - 2.79	2	2.85-2.92	13	2.68-3.12
	ycf4+psbD	3	2.66 - 3.08	5	2.69-3.12	6	2.61-2.99
	ycf4+psbE	5	2.59 - 3.01	6	2.66-3.16	6	2.72-3.17
**Ribosomal proteins** **/RNA**	ycf4+rps2	5	2.63 - 2.88	7	2.70-3.30	14	2.64-3.04
	ycf4+rps16	3	2.81 - 3.08	11	2.45-3.22	18	2.52-3.27
	ycf4+rrn16	10	2.63-3.19	6	2.72-3.18	18	2.55-3.29
**ATP** **synthase**	ycf4+atpB	12	2.56 - 3.15	8	2.62-3.12	28	2.54-3.27
	ycf4+atpI	zero	–	3	2.88-3.27	7	2.69-3.25
**Other genes**	ycf4+ycf10	5	2.57 - 3.33	1	2.85	2	2.69, 2.79
	ycf4+rbcL	13	2.56 - 3.15	8	2.75-3.31	17	2.54-2.98
	Ycf4+rbcS	10	2.75-3.10	9	2.64-3.22	14	2.58-2.96
	ycf4+clpP	6	2.52 - 2.99	6	2.63-2.92	13	2.64-3.22
	ycf4+rpoA	12	2.45 - 3.34	7	2.72-3.09	12	2.64-3.24
	ycf4+rpoB	9	2.64 - 3.29	9	2.65-3.33	25	2.62-3.23
	ycf4+accD	10	2.62 - 3.29	4	2.70-3.29	7	2.71-2.88
	ycf4+petA	5	2.88 - 3.22	5	2.71-2.83	10	2.63-3.00

## Discussion

The photosystem I complex requires both plastid and nuclear-encoded proteins for biosynthesis. PS1 assembly requires the association of several redox cofactors, chromophores, and Fe-S clusters. Ycf4 is a crucial auxiliary element in the PS1 assembly process. It has previously been shown to be important in photosystem I (PSI) formation in the unicellular green alga *Chlamydomonas reinhardtii*, with ycf3- and YCF4-deficient mutants unable to develop photoautotrophically and accumulate PSI (Boudreau et al., 1997). They concluded that Ycf3 and Ycf4 are not essential for PSI subunit synthesis but are most likely involved in PSI complex assembly. Orf184 mutants of *Cyanobacterium synechocystis* grew normally like wild-type cells, according to Wilde et al. (1995). However, the pigment content of mutant cells (especially the phycocyanin to chlorophyll ratio) differed significantly from that of wild-type cells. Another study found that YCF4 mutants could maintain photoautotrophic growth. They believed the YCF4 gene product was not necessary for photosynthesis. Despite the fact that mutants had lower PSI levels, this was not due to a deficit in plastid gene expression.

They concluded that Ycf3 and Ycf4 are not required for the synthesis of PSI subunits but are most likely involved in the assembly of the PSI complex. However, pigment composition (particularly phycocyanin to chlorophyll ratio) of mutant cells was distinctly different from those of wild-type cells. Another research group also concluded that YCF4 mutants were able to sustain photoautotrophic growth. They were of the view that the YCF4 gene product is not essential for photosynthesis. Though mutants were deficient in PSI contents, this deficiency was not caused by a defect in plastid gene expression. Rather, it was suggested that YCF4 plays a key role at the post-translational stage, resulting in faulty PSI assembly or reducing its stability (Krech et al., 2012).

Contrarily, we establish that tobacco homoplastic *YCF4* mutants are heterotrophic and cannot survive in an autotrophic environment. Carbon starvation appeared to inhibit plant growth, as mutants were unable to grow on MS medium containing sucrose up to 10 mg/L. Plants appeared to survive at 15-30 mg/L, although their growth was limited. When switched to compost-containing pots, these plants were photosynthetically incompetent and could not grow photoautotrophically. Our findings contradict that of Krech et al. (2012), who claimed that photoautotrophic growth was possible in YCF4 mutant plants.

Our findings, however, are consistent with those of Boudreau et al. (1997), who knocked out the nearly entire *YCF4* gene from *C. reinhardtii* and concluded that the *YCF4* gene product is essential for photosynthesis. The expression of the plastid-encoded genes encoding for the key subunits of photosystem I (*psaA*, *psaB*, *psaC*, and *psaH*) and Photosystem-II (*psbA*, *psbB*, *psbC*, *psbD*, *psbE*) were unaffected in *YCF4* plants, indicating that Ycf4 deletion did not appear to have any direct role in photosystem biogenesis. However, *YCF4* plants had much lower levels of *lhc* (light-harvesting complex) and *rbcL* (large subunit of ribulose-1,5-bisphosphate carboxylase-oxygenase) expression than wild-type normal plants. The LHC is important for the formation of a super complex photosystem (PSI) whereas rbcL is critical for RUBISCO. Decreased expression of LHC and rbcL may affect the conformation of the photosystem and accumulation of functional RUBISCO respectively, resulting in defective photosynthesis.

In the knockout plants, the microscopic studies revealed that chloroplast structure was abnormal, presumably due to the lack of YCF4 protein. Wild-type chloroplasts were substantially larger and oblong in shape, but knockout chloroplasts were much smaller and spherical. The thylakoid membranes appeared to be less organized with certain vesicular structures. Chloroplasts of non-green senescing Broccoli florets have shown similar disorganization and subsequent formation of vesicular structure (Terai and Watada, 2000) that has been attributed to the disorganization and disintegration of thylakoid membranes.

The size of the deleted *YCF4* section may be a fundamental difference in the performance of *YCF4* tobacco mutants generated by Krech et al. (2012) and mutants reported in the current investigations. The carboxyl terminus spanning 91 amino acids of the YCF4 protein had not been knocked out, and the resultant mutants were able to grow photoautotrophically whereas, we have knocked out the entire open reading frame and found that knocked plants could not survive photoautotrophically and grew stuntedly at 30 g/L sucrose. The results of the *in-silico* studies supported our findings since the interactions between the photosystem-I subunits psaB, psaC, psaH, and LHC and the carboxyl terminus of the YCF4 were stronger than those between the amino and carboxyl termini. Similar to this, the interaction of YCF4 with the large (encoded by chloroplast) and small (encoded by nuclear) subunits of RuBicCO also supported the significance of the carboxyl terminus. Hence, this study is direct evidence of the fact that deletion of full-length YCF4 has made the plants unable to survive photo-autotrophically as interaction with all the proteins engaged directly or indirectly in photosynthesis has revealed strong binding patterns with the carboxyl terminus of YCF4.

We came to the conclusion that deletion of the entire YCF4 open reading frame prevents tobacco plants from growing autotrophically, even though photosystem biogenesis is a complex process and various co-factors and related proteins still need to be investigated. Under heterotrophic circumstances (30g/L sucrose), plants could thrive, but they grew stuntedly and died when placed in pots with peat moss.

## Data availability statement

The original contributions presented in the study are included in the article/**Supplementary Materials**. Further inquiries can be directed to the corresponding author.

## Author contributions

MK conceived the idea, supervise the students, and wrote the manuscript. MM, RR, KM, and GM performed data analysis. Whereas, FJ handled microscopic studies and helped in the manuscript write-up. All authors contributed to the article and approved the submitted version.

## Funding

Funds were provided by the Ministry of Science & Technology, Islamabad Pakistan to MK.

## Acknowledgments

The authors are highly thankful to the Ministry of Science & Technology, Islamabad Pakistan.

## Conflict of interest

The authors declare that the research was conducted in the absence of any commercial or financial relationships that could be construed as a potential conflict of interest.

## Publisher’s note

All claims expressed in this article are solely those of the authors and do not necessarily represent those of their affiliated organizations, or those of the publisher, the editors and the reviewers. Any product that may be evaluated in this article, or claim that may be made by its manufacturer, is not guaranteed or endorsed by the publisher.

## Abbreviations

YCF4, Hypothetical chloroplast open reading frame; PSI, Photosystem I; PSII, Photosystem II; Cytb_6_f, cytochrome b_6_f complex.
