# Supplementary material for: The tobacco chloroplast YCF4 gene is essential for transcriptional gene regulation and plants photoautotrophic growth
Source: Front Plant Sci. 2022 Oct 24;13:1014236. doi: 10.3389/fpls.2022.1014236 (PMC9638951; doi:10.3389/fpls.2022.1014236)
Supplement: Supplementary file 1 [file DataSheet_1.pdf]

# *Supplementary Material*

## 1 Supplementary Figures

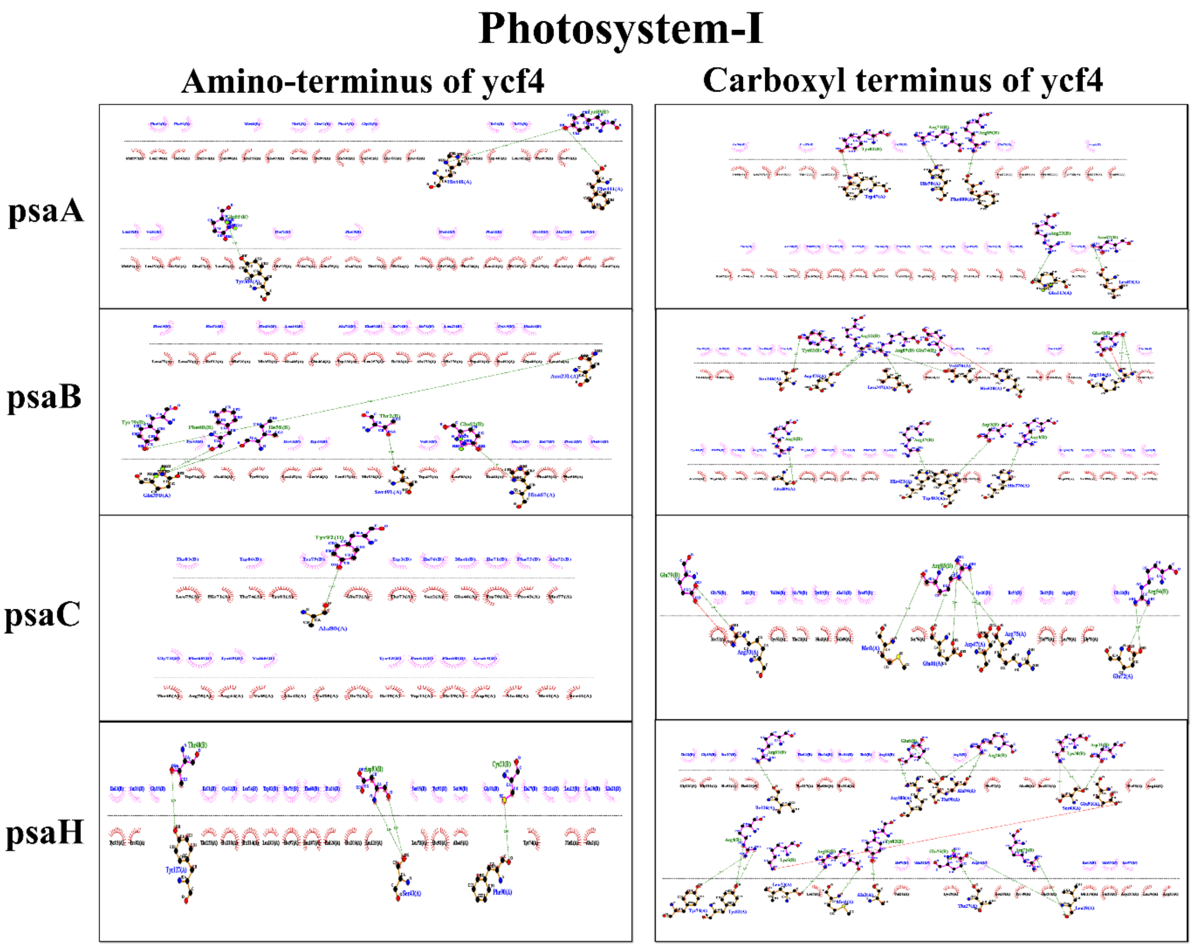


**Supplementary Figure 1.** The molecular interaction of photosystem-I proteins (psaA, psaB, psaC and psaH) with amino-terminus and carboxyl-terminus of ycf4 protein.

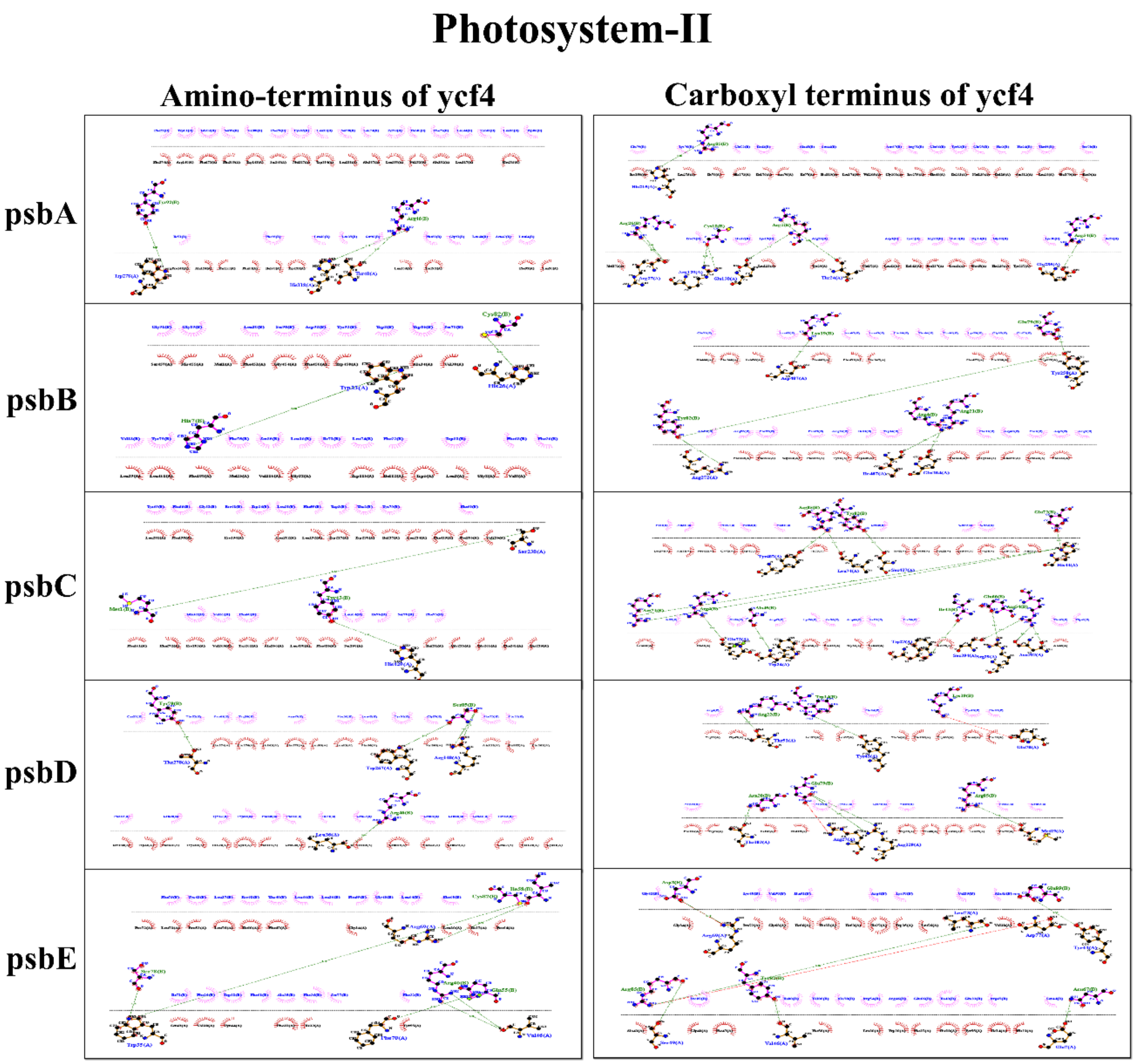


**Supplementary Figure 2.** The molecular interaction of photosystem-II proteins (psbA, psbB, psbC, psbD and psbE) with amino-terminus and carboxyl-terminus of ycf4 protein.

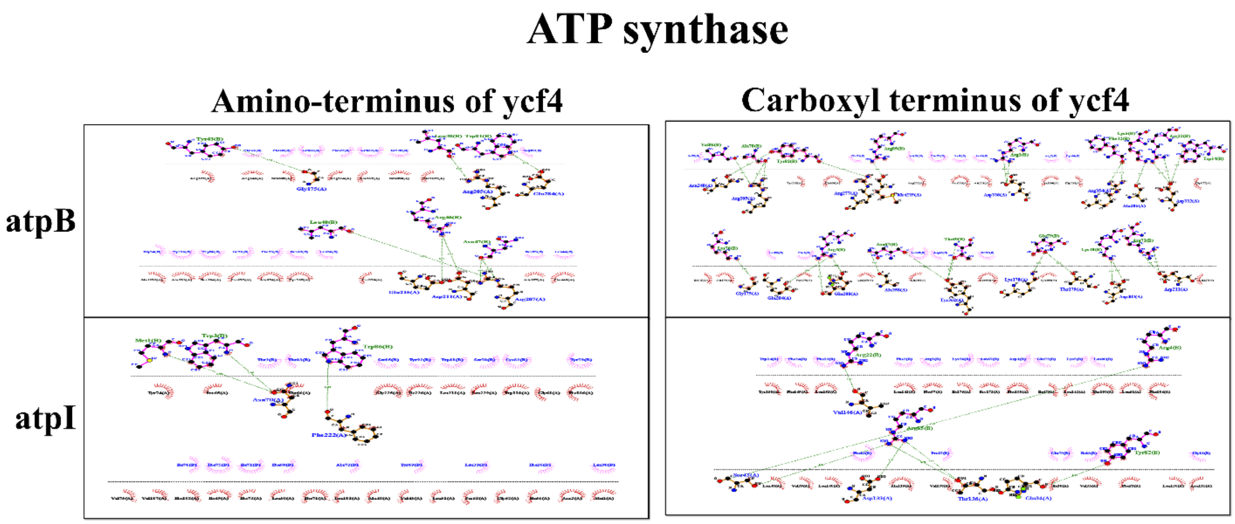


**Supplementary Figure 3.** The molecular interaction of subunits of ATP synthase (atpB and atpI) with amino-terminus and carboxyl-terminus of ycf4 protein.

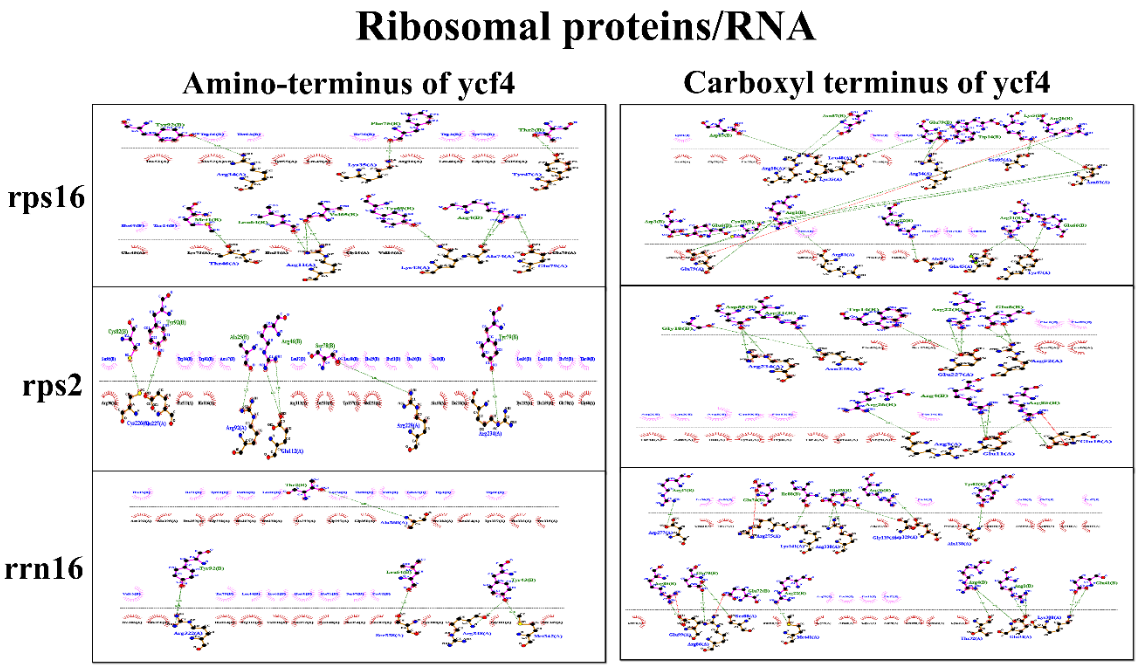


**Supplementary Figure 4.** The molecular interaction of ribosomal proteins/RNA (rps16, rps2 and rrn16) with amino-terminus and carboxyl-terminus of ycf4 protein.

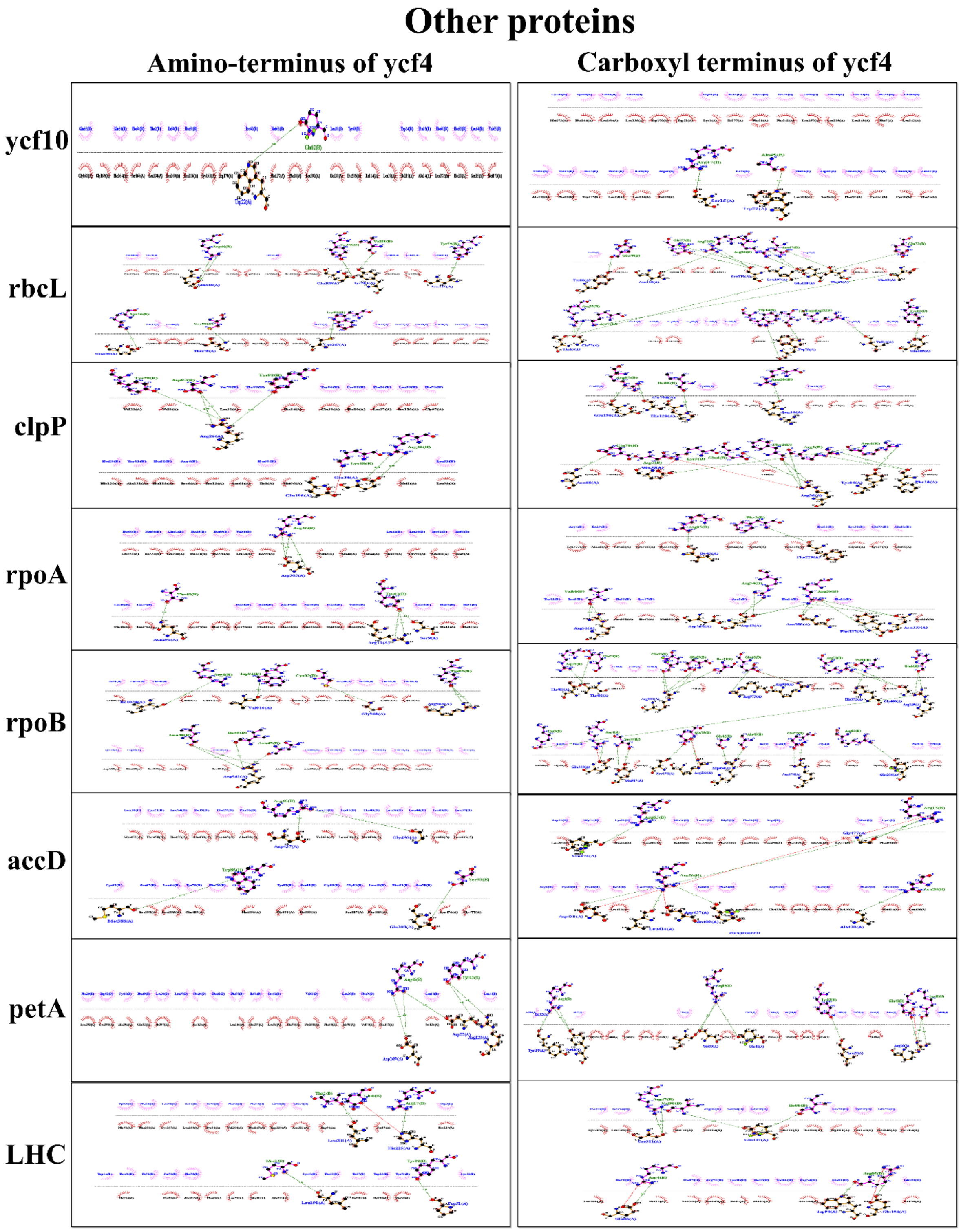


**Supplementary Figure 5.** The molecular interaction of other proteins (ycf10, rbcL, clpP, rpoA, rpoB, accD, petA and LHC) with amino-terminus and carboxyl-terminus of ycf4 protein.
